# Tetrandrine inhibits migration and invasion of human renal cell carcinoma by regulating Akt/NF-κB/MMP-9 signaling

**DOI:** 10.1371/journal.pone.0173725

**Published:** 2017-03-13

**Authors:** Shurui Chen, Wei Liu, Ke Wang, Yizeng Fan, Jiaqi Chen, Jianbin Ma, Xinyang Wang, Dalin He, Jin Zeng, Lei Li

**Affiliations:** 1 Department of Urology, the First Affiliated Hospital of Xi'an Jiaotong University, Xi'an, PR China; 2 Department of Science and Technology, Jinzhou Medical University, Jinzhou, PR China; University of South Alabama Mitchell Cancer Institute, UNITED STATES

## Abstract

Renal cell carcinoma (RCC) is known as one of the most lethal malignancies in the urological system because of its high incidence of metastasis. Tetrandrine (Tet), a traditional Chinese herbal medicine, exerts a potent anti-cancer effect in a variety of cancer cells. However, the anti-metastatic effect of Tet and its possible mechanism in RCC is still unclear. The present study revealed that Tet significantly suppressed the migration and invasion of RCC 786-O and 769-P cells *in vitro*. Mechanistically, the protein levels of matrix metalloproteinases 9 (MMP-9), phosphorylated PI3K, PDK1, Akt and NF-κB were markedly reduced after Tet treatment. Moreover, co-treatment with LY294002 (PI3K inhibitor) could further enhance the Tet-inhibited migration and invasion, and the NF-κB and MMP-9 protein levels were further decreased. Similar results were observed after PDTC (NF-κB inhibitor) co-treatment. Conversely, SC79, an Akt activator, could partially reverse the anti-metastatic effects of Tet, accompanied by the restoration of NF-κB and MMP-9 protein levels. In conclusion, the current results indicated that Tet inhibited migration and invasion of RCC partially by regulating Akt/NF-κB/MMP-9 signaling pathway, suggesting that Tet may be a potential therapeutic candidate against metastatic RCC.

## Introduction

Renal cell carcinoma (RCC) is the third most common urologic malignancy, accounting for approximately 3% of all cancers and 90% of kidney cancers in adults [1]. The advanced diagnostic methods and systemic treatment (chemotherapy and radiotherapy), benefits a remarkable improvement in 5-year survival and recurrence-free survival. Despite that, 30% patients with RCC continue to progress into the metastatic disease, resulting in a median overall survival of metastatic RCC of only 12 months [2]. Furthermore, it is insensitive to chemotherapy, and drugs for immunotherapy and targeted therapy have a low effect on the treatment for metastatic RCC [3]. Thus, there is an urgent need to explore novel therapeutic agents against metastatic RCC.

Tumor metastasis has been identified as a leading cause of cancer-related deaths [4]. Moreover, the degradation of the extracellular matrix (ECM) by proteinases is a vital step in the metastatic process [5]. Matrix metalloproteinases (MMPs), members of the zinc-dependent endopeptidases, could modulate the breakdown of the ECM and facilitate tumor invasion [6, 7]. Among the MMPs, MMP-9 is closely correlated with tumor metastasis in various cancers, including gastric cancer [8], prostate cancer [9], and cervical cancer [10].Therefore, MMP-9 and other MMPs may be considered as effective targets for anti-cancer drugs [11].

Tetrandrine (Tet), a bisbenzylisoquinoline alkaloid, is isolated from traditional Chinese medicine Stephaaniae [12]. Tet has been widely used as anti-hypertension, anti-arrhythmic, and anti-rheumatism agent [13–15]. Recently, accumulating evidence suggested the anti-cancer effects on various cancers, which were associated with growth inhibition, induction of apoptosis and cell cycle arrest, and suppression of angiogenesis *in vitro* and *in vivo* [16, 17]. Our previous study had demonstrated the anti-cancer effects of Tet on the bladder and prostate cancers [18, 19]. Despite its potential of anti-proliferation in solid tumors, whether Tet inhibits cell migration and invasion of RCC has not yet been elucidated. Also, the underlying mechanism of Tet on cell migration and invasion is unknown. Hence, our study aimed to explore the effects of Tet on RCC cell lines and to investigate its possible mechanisms.

## Material and methods

### Cell culture

Human RCC cell lines 786-O and 769-P were obtained from the American Type Culture Collection (ATCC; Manassas, VA, USA) and cultured in RPMI 1640 medium, which contains 10% fetal bovine serum (FBS; Gibco, Grand Island, NY, USA) and 1% penicillin-streptomycin(Invitrogen, Carlsbad, CA, USA), in a humidified incubator with 5% CO_2_ at 37°C.

### Reagents

Tetrandrine (Tet) (C_38_H_42_N_2_O_6_) and 3-(4,5-dimethylthiazol-2-yl)-2,5-diphenyltetrazolium bromide (MTT) were obtained from Sigma Chemical Co. (St. Louis, MO, USA). Tet was solubilized in 0.1 M HCl to a concentration of 25 mg/mL as the stock solution and then diluted to the desired concentrations before use. Antibodies against PI3K, phosphor-PI3K, PDK1, p-PDK1, Akt, phospho-Akt, NF-κB, and MMP-9 were purchased from Cell Signaling Technology, Inc. (Beverly, MA, USA). LY294002 (PI3K inhibitor), PDTC (NF-κB inhibitor), TNF-α (NF-κB activator) were purchased from Santa Cruz Biotechnology, Inc. (Santa Cruz, CA, USA). SC79 were purchased from abcam. Inc.(Cambridge, Britain).

### MTT assay

786-O and 769-P cells were seededin 96-well plates (1×10^4^ cells/well, 90% density) and exposed to different doses of Tet. Then, 0.5 mg/mL MTT dye solution was added to each well, and the cells were incubated at 37°C for 4 h. Subsequently, the culture medium was discarded, and dimethyl sulfoxide (DMSO) was added to solubilize the precipitate. A 96-well microplate reader (Bio-Rad, Hercules, CA, USA) was used to estimate the absorbance at 490 nm.

### Wound healing assay

RCC 786-O or 769-P cells were seeded in 6-well plates. When the cell density reached up to 90% confluency, the cell monolayer was scratched using a 200-μL pipette tip. Then, the wounded cells were treated with Tet at different times and visualized in six randomly chosen fields by microscopy to evaluate the ability of cell migration.

### Transwell migration assay

Transwell migration assays were performed to detect the anti-migratory ability of Tet on human RCC 786-O and 769-P cells. The cells (786-O: 2×10^4^ or 769-P: 3×10^4^ per chamber, respectively) treated with or without Tet were seeded into the upper chamber, while 800 μL of the medium containing 10% fetal calf serum was added to the lower chamber. After incubation in a humidified atmosphere at 37°C for 24 h, the non-migrated cells in the upper chamber were removed with a cotton swab. The migrated cells in the bottom chamber were fixed with 4% paraformaldehyde for 10 min, stained with 0.1% crystal violet for 10 min, and images captured with a microscopy five randomly chosen fields at 100× magnification.

### Matrigel invasion assay

The upper parts of the transwell apparatus (polycarbonic membrane, 6.5 mm diameter, 8 μm pore size) were coated with 50 μL of 1:5 mixture of Matrigel: RPMI1640 medium. Cells (786-O: 8×10^4^ or 769-P: 8×10^4^ per chamber, respectively) treated with Tet were seeded into the upper chamber and incubated at 37°C for 4 h. The subsequent procedure was similar to the transwell migration assay.

### Western blotting

The RCC cells were collected and lysed after the Tet treatment. The clarified protein lysates (approximately 30–60 μg) were electrophoresed on SDS-polyacrylamide gel (10%) and transferred to polyvinylidene fluoride membranes. Western blotting was performed with primary indicated antibodies at 4°C, overnight. Then, the membranes were washed with TBST buffer and incubated with horseradish peroxidase-conjugated secondary antibody for 1 h. Subsequently, the proteins of interest were analyzed by enhanced chemoluminescence and quantified using Image Lab software (Bio-Rad, Hercules, CA, USA).

### Statistical analysis

GraphPad Prism 5.2 software (GraphPad Software Inc., La Jolla, CA, USA) was used for all statistical analyses. Statistical differences in different groups were compared by Student’s *t*-test (two-sided) or one-way analysis of variance (ANOVA). Significant differences were represented as *P*<0.05.

## Results

### Effects of Tet on cell proliferation of RCC 786-O and 769-P cells

**Fig 1:**
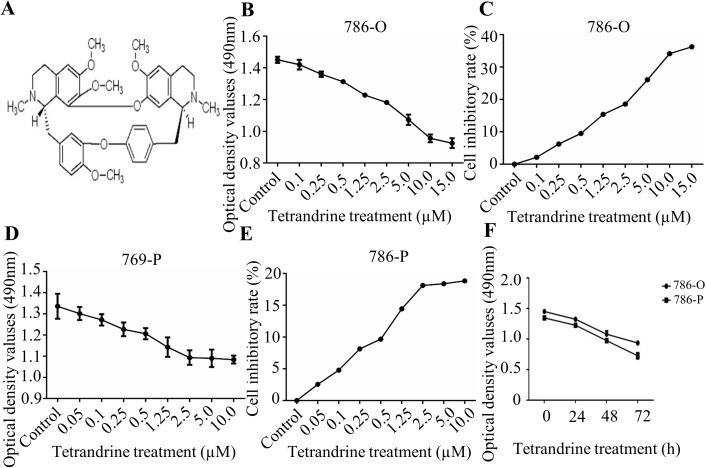
Tetrandrine (Tet) inhibits human RCC proliferation **A.** The chemical structure of Tet. The OD value and inhibition rate of Tet on viability of RCC 786-O (**B, C**) and 769-P (**D, E**) cells. Cells under 90% cell density were treated with the vehicle control or Tet at the indicated concentrations for 24h. The dynamic OD value was assayed at the concentration of 0.5 μM (**F**). All the cell viability was detected by modified MTT assay. The values were showed as mean±S.E. The experiment was performed in triplicate.

Before we explored the biological effects of Tet (Fig 1A) on cell mobility ability, we first investigated the effects of Tet on cell growth using a modified MTT assay. The 786-O and 769-P cells with 90% density were seeded and treated with various concentrations of Tet from 0.05 to 15.0 μM (Fig 1B and 1D). And the effects of Tet on 786-O cell growth inhibition, was shown in Fig 1C, with the 24h Tet treatments at 0.1 μM, 0.25 μM and 0.5 μM resulting in 2.2%, 6.3%, and 9.5% of inhibitory rate, respectively. Similar effects were also exhibited in 769-P cells (Fig 1E).The results indicated that Tet markedly inhibited cell growth at the concentration of ≥0.5 μM in these two cell lines, while Tet at 0.5 μM represented for a less than 10% inhibitory rate of cell proliferation. Furthermore, a time-dependent manner of cell growth inhibition was observed in 786-O and 769-P cells after Tet treatment (Fig 1F). Based the results from the MTT assay, Tet at 0.5 μM was chosen to conduct the subsequent experiment.

### Effects of Tet on migration and invasion of RCC 786-O and 769-P cells

**Fig 2:**
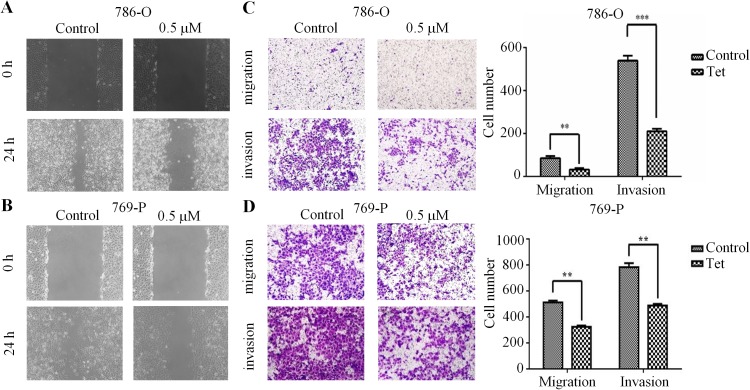
Tet suppresses cell migration and invasion of human RCC. The inhibition of Tet on 786-O (**A**) and 769-P (**B**) cells were determined by wound closure assay. The width of scratch was measured in vehicle or Tet group. 786-O (**C**) and 769-P (**D**) cells were treated with vehicle or Tet for 24 h using the Transwell migration and invasion assay. The quantitative data were shown in the right panel. The values were showed as mean ± S.E. All the experiments were performed in triplicate (** means *P*<0.05, *** means *P*<0.01).

To further analyze whether Tet could inhibit the migration and invasion of human RCC, we performed wound healing assay and transwell assay. Results from wound healing assay indicated that 0.5 μM Tet-treated 786-O cells migrated much more slowly compared with control cells at 24 h (Fig 2A). Similarly, 769-P cells treated with Tet also greatly impeded cell migration (Fig 2B). Additionally, we also found that Tet markedly attenuated the migration and invasion of 786-O and 769-P cells using a transwell assay (Fig 2C and 2D). Altogether, these results confirmed the ant-metastatic effect of Tet on RCC cells, as determined by wound healing assay and transwell assay.

### Tet significantly decreased the expressions of NF-κB, MMP-9, phospho-AKT, phospho-PI3K and phospho-PDK1 in human RCC

**Fig 3:**
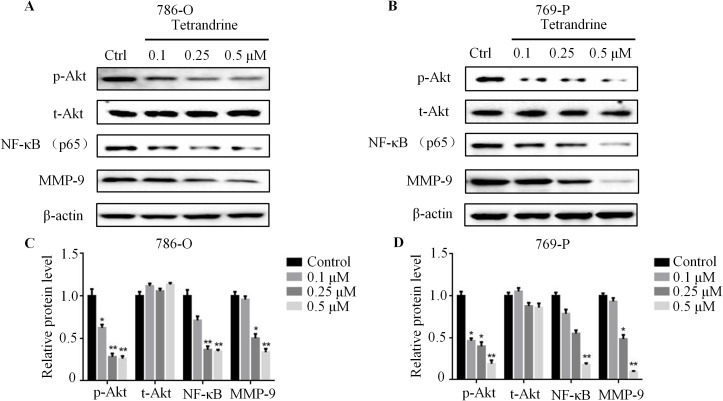
Tet markedly decreases phospho-AKT, NF-κB and MMP-9 expression in human RCC. 786-O (**A**) and 769-P (**B**) cells treated with vehicle or Tet (0.1μM to 0.5μM) for 24 h were immunoblotted for the expression of AKT, phospho-AKT, NF-κB and MMP-9. β-actin was used as a loading control. The quantitative data were shown in the lower panel (**C,D**). The values were showed as mean±S.E. Representative results from three independent experiments were shown (** means *P*<0.05, *** means *P*<0.01).

Previous studies have reported that Akt/NF-κB signaling pathway was positively correlated with tumor metastasis [20, 21]. To explore whether Akt/NF-κB signaling was also necessary for Tet-induced anti-metastatic effects on 786-O and 769-O cells, Western blotting was then performed. As expected, the protein level of NF-κB was markedly reduced by Tet in a concentration-dependent manner (Fig 3A–3D). Additionally, exposing 786-O and 769-P cells to Tet resulted in a significant suppression of Akt phosphorylation. Intriguingly, the levels of phospho-PI3K and phospho-PDK1 protein, which were upstream regulators of Akt, were also decreased after Tet treatment, suggesting a role of Tet in inactivation of Akt/NF-κB signaling pathway (Fig 3A–3D and S1 Fig). Previous studies have reported that MMP-9, an indicator of tumor metastasis, was regulated by NF-κB [9]. Our results exhibited that there was a dramatic decrease of MMP-9 expression in Tet-treated 786-O and 786-P cells (Fig 3A–3D). Taken together, these results indicated that Akt/NF-κB/MMP-9 signaling was inhibited in Tet-treated 786-O and 769-P cells.

### Akt phosphorylation was involved in Tet-induced anti-metastatic effects in RCC

**Fig 4:**
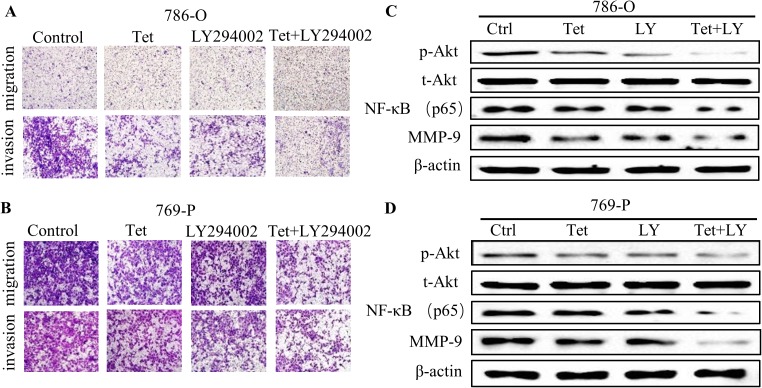
Tet inhibits RCC migration and invasion *via* decreasing Akt, NF-κB and MMP-9 expression. The metastatic effects of 786-O (**A**) and 769-P (**B**) were detected by transwell assay under Tet treatment (0.5μM), with or without LY294002 (LY, 20μM) for 24 h. The protein levels of Akt, phospho-Akt, NF-κB and MMP-9 in 786-O (**C**) and 769-P (**D**) cells were detected after indicated treatments by western blotting. And the experiments were performed in triplicate.

To further explore the role of Akt in Tet-inhibited cell migration and invasion, LY294002 (PI3K inhibitor), which was widely used to inhibit the activation of Akt, was used to combine with Tet for the subsequent experiment. Our results revealed that co-treatment with LY294002 could further reinforce the anti-metastatic effects of Tet in 786-O and 769-P cells (Fig 4A and 4B), as well as the protein levels of NF-κB and MMP-9 compared with the Tet alone and LY294002 alone treatment cells (Fig 4C and 4D). Additionally, the Akt activator SC79, could remarkably reverse the inhibitory effect of migration and invasion by Tet in RCC, accompanied with the restoration of NF-κB and MMP-9 protein levels (S2A–S2D Fig). These data strongly supported that Tet exhibited anti-metastasis effect by inhibition of Akt phosphorylation.

### Tet inhibited human RCC migration and invasion via negatively regulating NF-κB expression

**Fig 5:**
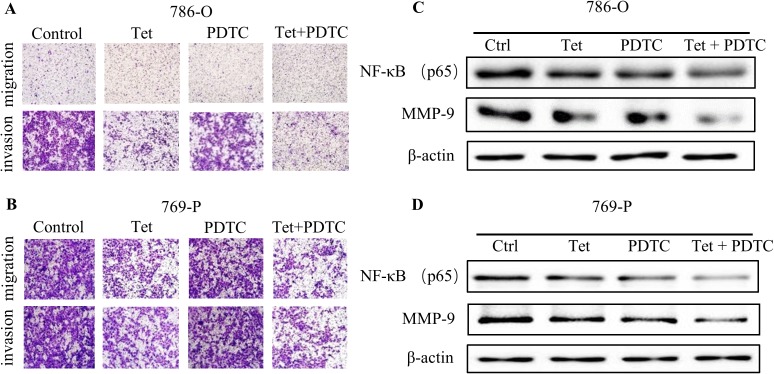
Tet represses cell migration and invasion of human RCC by negatively regulating NF-κB expression. The metastatic phenotype of 786-O and 769-P was determined by transwell assay. The migrated and invaded 786-O (**A)** and 769-P (**B**) cells were counted after treatment with Tet (0.5μM), PDTC (10μM), or the both for 24 h. Under similar treatment, lysates from 786-O (**C**) and 769-P (**D**) cells treated with Tet or PDTC were immunoblotted for MMP-9 and NF-κB. All the experiments were performed in triplicate. Representative results from three independent experiments were shown.

To gain deeper insights into the involvement of Akt/ NF-κB signaling, PDTC, a NF-κB inhibitor, was then used. As expected, the anti-metastatic effect of Tet on RCC could be further enhanced by PDTC (Fig 5A and 5B), and a dramatic decline of MMP-9 protein levels was observed (Fig 5C and 5D), suggesting that NF-κB might function as a downstream regulator of Akt in Tet-inhibited cell migration and invasion.

## Discussion

Metastasis is a vast obstacle for systemic treatment in patients with RCC [22]. Metastasis is the hallmark of cancer, which spreads the tumors from the original site to the distant organs. Moreover, it is a complicated process implicated in local invasion, transfer, extravasation, and tumor deposit [23].Of the metastatic process, the degradation of ECM is a key step in initiating tumor invasion, and interference with this step may be an efficient strategy for tumor treatment [24]. For tumor invasion and metastasis, MMPs are the crucial proteolytic enzymes to degrade the ECM and initiate the metastatic process [7]. Although more than one MMPs have been identified, MMP-9 is the enzyme most closely correlated with the degradation of ECM and is highly expressed in metastatic cancer [25].

Tet, a Chinese herbal medicine, exerts anti-metastasis activity in a variety of cancers. Our previous study revealed that Tet could inhibit the expression of MMP-9 *via* the inactivation of Akt/NF-κB signaling [9]. Additionally, Tet inhibited the metastasis of breast cancer *in vivo*, partially by regulating the endothelial cell-specific molecule-1 (ESM-1) and intercellular cell adhesion molecule-1 (ICAM-1) [26].In the present study, our findings revealed that Tet significantly suppressed the migratory and invasive abilities of human RCC 786-O and 769-P cells. Moreover, our results demonstrated that Tet could markedly inhibit the protein level of MMP-9 in both 786-O and 769-P cells. The anti-invasive capacity of Tet may be of great importance in decreasing cancer mortality in RCC patients. Further studies are needed to explore the underlying mechanism of Tet on the regulation of MMP-9.

NF-κB, a pivotal transcriptional factor in cancer cells, participates in the tumor progression by regulating angiogenesis and suppressing apoptosis [27, 28]. Also, it has been proven as a regulator of MMP-9 to promote tumor invasion and metastasis [29, 30]. In addition, Akt signaling regulates the expression of NF-κB as well as MMP-9 in several cancer cells [20, 21]. Thus, to elucidate the effect of Tet on activation, Akt/NF-κB signaling was assessed by western blotting. In our study, Akt, PI3K, PDK1 phosphorylation and NF-κB activation were found to be inhibited by Tet treatment, whereas, no remarkable effect was observed on total Akt, PI3K and PDK1, indicating that Tet could inhibit Akt/NF-κB signaling pathway in 786-O and 769-P cells.

Moreover, PI3K inhibitor LY294002 further reinforced the decrease of NF-κB and MMP-9 protein levels induced by Tet. Similar results were observed after NF-κB inhibitor PDTC treatment. In contrast, the decreased NF-κB and MMP-9 protein levels by Tet were partially reversed after SC79 treatment. These findings strongly suggested that Tet exerted an anti-metastatic effect at least partially by Akt/NF-κB/MMP-9 axis.

However, our present study has several limitations. Although, we confirmed that Akt/NF-κB signaling pathway played an essential role in Tet-inhibited cell migration and invasion, the effect of the downstream molecules of Akt, such as activator protein-1 (AP-1) and STAT3 were still unknown. On the other hand, we did not establish a lung metastasis *in vivo* model of nude mice to validate our *in vitro* results. Therefore, further investigations are imperative to resolve these issues.

Our results concluded that Tet suppressed cell migration and invasion of human RCC by negatively regulating Akt/NF-κB signaling and MMP-9 expression. Importantly, our findings suggest a potentially significant correlation of Tet and Akt/NF-κB signaling in the metastatic progression of human RCC; therefore, Tet may be a potential chemotherapeutic candidate against metastatic RCC.

## Supporting information

S1 Fig Effects of Tet on the phosphorylated levels of PI3K and PDK1. 786-O (**A**) and 769-P (**B**) cells treated with vehicle or Tet (0.1μM to 0.5μM) for 24 h were immunoblotted for PI3K, phospho-PI3K, PDK1 and phospho-PDK1. β-actin was used for a loading control. Representative results from three independent experiments were shown.
(TIF)

S2 Fig Akt activator SC79 reverses Tet-induced anti-metastatic effects. *In vitro* cell mobility of 786-O (**A**) and 769-P (**B**) cells were determined by transwell assay under tetrandrine (0.5μM), SC79 (10μM), or the combined treatment for 24 h. The protein levels of Akt, phospho-Akt, NF-κB and MMP-9 of 786-O (**C**) and 769-P (**D**) cells were detected as the indicated treatments by western blotting after SC79 treatment. Representative results from three independent experiments were shown.
(TIF)

## References

[1] Comperat E, Camparo P. Histological classification of malignant renal tumours at a time of major diagnostic and therapeutic changes. Diagnostic and interventional imaging. 2012;93(4):221–31. 10.1016/j.diii.2012.01.015 22465787

[2] Cohen HT, McGovern FJ. Renal-cell carcinoma. The New England journal of medicine. 2005;353(23):2477–90. 10.1056/NEJMra043172 16339096

[3] Duensing S, Hohenfellner M. Adjuvant therapy for renal-cell carcinoma: settled for now. Lancet. 2016;387(10032):1973–4. 10.1016/S0140-6736(16)00653-X 26969091

[4] Hainaut P, Plymoth A. Targeting the hallmarks of cancer: towards a rational approach to next-generation cancer therapy. Current opinion in oncology. 2013;25(1):50–1. 10.1097/CCO.0b013e32835b651e 23150341

[5] Yilmaz M, Christofori G, Lehembre F. Distinct mechanisms of tumor invasion and metastasis. Trends in molecular medicine. 2007;13(12):535–41. 10.1016/j.molmed.2007.10.004 17981506

[6] Zhou F, Liu D, Ning HF, Yu XC, Guan XR. The roles of p62/SQSTM1 on regulation of matrix metalloproteinase-9 gene expression in response to oxLDL in atherosclerosis. Biochem Biophys Res Commun. 2016;472(3):451–8. 10.1016/j.bbrc.2016.01.065 26898796

[7] Kessenbrock K, Plaks V, Werb Z. Matrix metalloproteinases: regulators of the tumor microenvironment. Cell. 2010;141(1):52–67. PubMed Central PMCID: PMC2862057. 10.1016/j.cell.2010.03.015 20371345 PMC2862057

[8] Chang X, Xu X, Xue X, Ma J, Li Z, Deng P, et al NDRG1 Controls Gastric Cancer Migration and Invasion through Regulating MMP-9. Pathology oncology research: POR. 2016;22(4):789–96. 10.1007/s12253-016-0071-8 27154576

[9] Kou B, Liu W, He W, Zhang Y, Zheng J, Yan Y, et al Tetrandrine suppresses metastatic phenotype of prostate cancer cells by regulating Akt/mTOR/MMP-9 signaling pathway. Oncology reports. 2016;35(5):2880–6. 10.3892/or.2016.4649 26935264

[10] Fan D, Wang Y, Qi P, Chen Y, Xu P, Yang X, et al MicroRNA-183 functions as the tumor suppressor via inhibiting cellular invasion and metastasis by targeting MMP-9 in cervical cancer. Gynecologic oncology. 2016;141(1):166–74. 10.1016/j.ygyno.2016.02.006 26873866

[11] Li C, Li F, Zhao K, Yao J, Cheng Y, Zhao L, et al LFG-500 inhibits the invasion of cancer cells via down-regulation of PI3K/AKT/NF-kappaB signaling pathway. PLoS One. 2014;9(3):e91332 PubMed Central PMCID: PMC3950212. 10.1371/journal.pone.0091332 24618693 PMC3950212

[12] Liu T, Liu X, Li W. Tetrandrine, a Chinese plant-derived alkaloid, is a potential candidate for cancer chemotherapy. Oncotarget. 2016. 10.18632/oncotarget.8315 PMC5130046 27027348

[13] Wang X, Yang Y, Yang D, Tong G, Lv S, Lin X, et al Tetrandrine prevents monocrotaline-induced pulmonary arterial hypertension in rats through regulation of the protein expression of inducible nitric oxide synthase and cyclic guanosine monophosphate-dependent protein kinase type 1. Journal of vascular surgery. 2015. 10.1016/j.jvs.2015.09.016 26527422

[14] Shen DF, Tang QZ, Yan L, Zhang Y, Zhu LH, Wang L, et al Tetrandrine blocks cardiac hypertrophy by disrupting reactive oxygen species-dependent ERK1/2 signalling. British journal of pharmacology. 2010;159(4):970–81. PubMed Central PMCID: PMC2829222. 10.1111/j.1476-5381.2009.00605.x 20105174 PMC2829222

[15] Gao LN, Feng QS, Zhang XF, Wang QS, Cui YL. Tetrandrine suppresses articular inflammatory response by inhibiting pro-inflammatory factors via NF-kappaB inactivation. Journal of orthopaedic research: official publication of the Orthopaedic Research Society. 2016;34(9):1557–68. 26748661 10.1002/jor.23155

[16] Zhang Y, Liu W, He W, Zhang Y, Deng X, Ma Y, et al Tetrandrine reverses epithelial-mesenchymal transition in bladder cancer by downregulating Gli-1. International journal of oncology. 2016;48(5):2035–42. 10.3892/ijo.2016.3415 26983576

[17] Lien JC, Lin MW, Chang SJ, Lai KC, Huang AC, Yu FS, et al Tetrandrine induces programmed cell death in human oral cancer CAL 27 cells through the reactive oxygen species production and caspase-dependent pathways and associated with beclin-1-induced cell autophagy. Environmental toxicology. 2016. 10.1002/tox.22238 26822499

[18] Li X, Su B, Liu R, Wu D, He D. Tetrandrine induces apoptosis and triggers caspase cascade in human bladder cancer cells. The Journal of surgical research. 2011;166(1):e45–51. 10.1016/j.jss.2010.10.034 21176918

[19] Liu W, Kou B, Ma ZK, Tang XS, Lv C, Ye M, et al Tetrandrine suppresses proliferation, induces apoptosis, and inhibits migration and invasion in human prostate cancer cells. Asian journal of andrology. 2015;17(5):850–3. PubMed Central PMCID: PMC4577603. 10.4103/1008-682X.142134 25677131 PMC4577603

[20] Chen S, Chen W, Zhang X, Lin S, Chen Z. Overexpression of KiSS-1 reduces colorectal cancer cell invasion by downregulating MMP-9 via blocking PI3K/Akt/NF-kappaB signal pathway. International journal of oncology. 2016;48(4):1391–8. 10.3892/ijo.2016.3368 26847533

[21] Chiu CT, Chen JH, Chou FP, Lin HH. Hibiscus sabdariffa Leaf Extract Inhibits Human Prostate Cancer Cell Invasion via Down-Regulation of Akt/NF-kB/MMP-9 Pathway. Nutrients. 2015;7(7):5065–87. PubMed Central PMCID: PMC4516987. 10.3390/nu7075065 26115086 PMC4516987

[22] Wang J, Zhao X, Qi J, Yang C, Cheng H, Ren Y, et al Eight proteins play critical roles in RCC with bone metastasis via mitochondrial dysfunction. Clinical & experimental metastasis. 2015;32(6):605–22. PubMed Central PMCID: PMC4503866. 26115722 10.1007/s10585-015-9731-4 PMC4503866

[23] Gupta GP, Massague J. Cancer metastasis: building a framework. Cell. 2006;127(4):679–95. 10.1016/j.cell.2006.11.001 17110329

[24] Dolle L, Depypere HT, Bracke ME. Anti-invasive/anti-metastasis strategies: new roads, new tools and new hopes. Current cancer drug targets. 2006;6(8):729–51. 17168676 10.2174/156800906779010263

[25] Park SY, Kim JH, Lee YJ, Lee SJ, Kim Y. Surfactin suppresses TPA-induced breast cancer cell invasion through the inhibition of MMP-9 expression. International journal of oncology. 2013;42(1):287–96. 10.3892/ijo.2012.1695 23151889

[26] Gao JL, Ji X, He TC, Zhang Q, He K, Zhao Y, et al Tetrandrine Suppresses Cancer Angiogenesis and Metastasis in 4T1 Tumor Bearing Mice. Evidence-based complementary and alternative medicine: eCAM. 2013;2013:265061. PubMed Central PMCID: PMC3677646. 23762115 10.1155/2013/265061 PMC3677646

[27] Yamaguchi N, Ito T, Azuma S, Ito E, Honma R, Yanagisawa Y, et al Constitutive activation of nuclear factor-kappaB is preferentially involved in the proliferation of basal-like subtype breast cancer cell lines. Cancer science. 2009;100(9):1668–74. 10.1111/j.1349-7006.2009.01228.x 19538528 PMC11158550

[28] Tai KY, Shieh YS, Lee CS, Shiah SG, Wu CW. Axl promotes cell invasion by inducing MMP-9 activity through activation of NF-kappaB and Brg-1. Oncogene. 2008;27(29):4044–55. 10.1038/onc.2008.57 18345028

[29] Kang H, Lee M, Choi KC, Shin DM, Ko J, Jang SW. N-(4-hydroxyphenyl)retinamide inhibits breast cancer cell invasion through suppressing NF-KB activation and inhibiting matrix metalloproteinase-9 expression. Journal of cellular biochemistry. 2012;113(9):2845–55. 10.1002/jcb.24159 22488409

[30] Thangapazham RL, Passi N, Maheshwari RK. Green tea polyphenol and epigallocatechin gallate induce apoptosis and inhibit invasion in human breast cancer cells. Cancer Biol Ther. 2007;6(12):1938–43. 18059161 10.4161/cbt.6.12.4974

