# Supplementary figures and images for: Tetrandrine inhibits migration and invasion of human renal cell carcinoma by regulating Akt/NF-κB/MMP-9 signaling
Source: PLoS One. 2017 Mar 13;12(3):e0173725. doi: 10.1371/journal.pone.0173725 (PMC5348026; doi:10.1371/journal.pone.0173725)

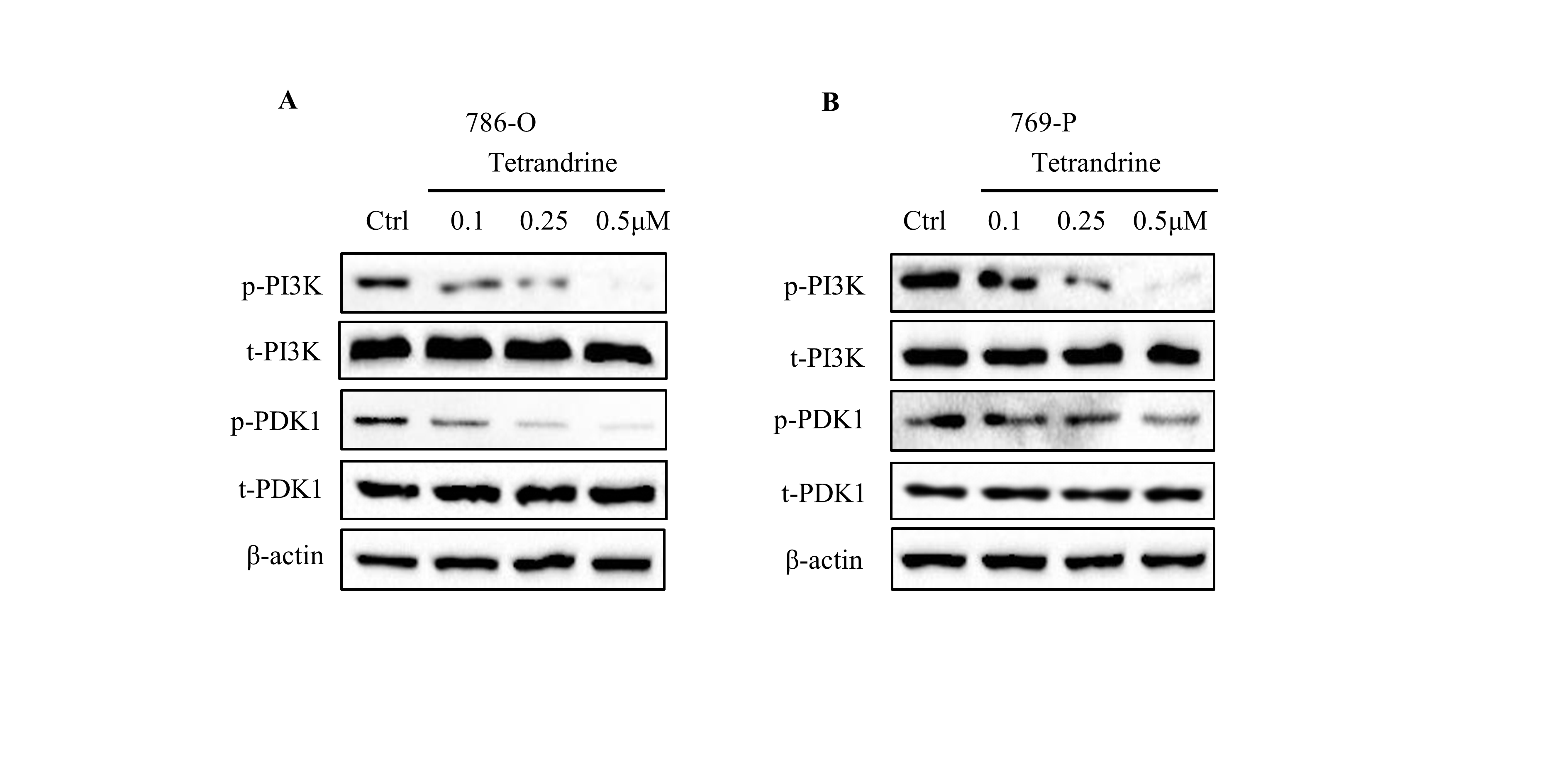

Supplement: S1 Fig — 786-O (A) and 769-P (B) cells treated with vehicle or Tet (0.1μM to 0.5μM) for 24 h were immunoblotted for PI3K, phospho-PI3K, PDK1 and phospho-PDK1. β-actin was used for a loading control. Representative results from three independent experiments were shown. (TIF) [file pone.0173725.s001.tif]

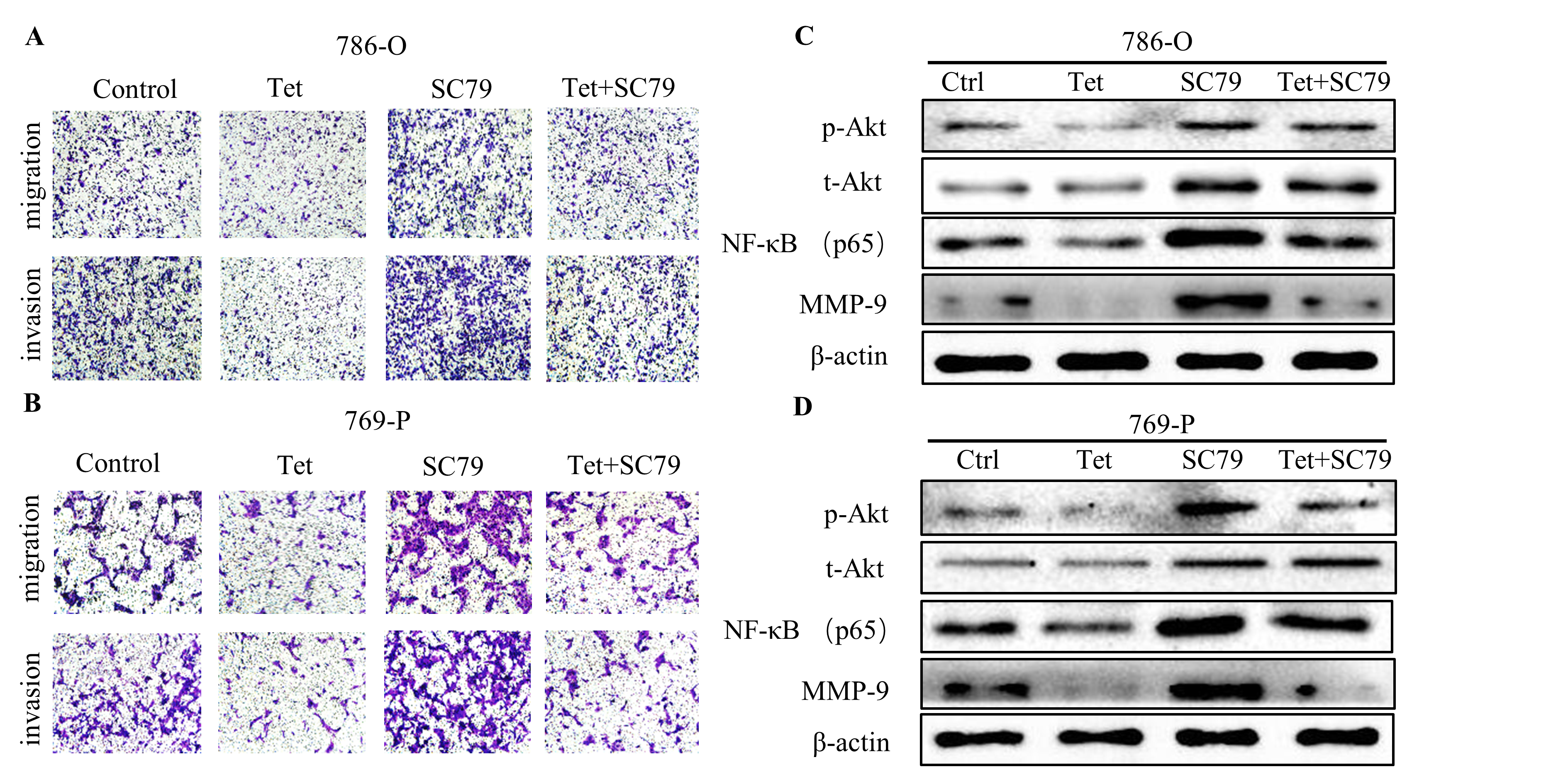

Supplement: S2 Fig — In vitro cell mobility of 786-O (A) and 769-P (B) cells were determined by transwell assay under tetrandrine (0.5μM), SC79 (10μM), or the combined treatment for 24 h. The protein levels of Akt, phospho-Akt, NF-κB and MMP-9 of 786-O (C) and 769-P (D) cells were detected as the indicated treatments by western blotting after SC79 treatment. Representative results from three independent experiments were shown. (TIF) [file pone.0173725.s002.tif]
